# Impact of a Labour Disruption Affecting Local Public Health on the Incidence of Chlamydia Infections in Toronto

**DOI:** 10.1371/journal.pone.0079375

**Published:** 2013-11-29

**Authors:** Andrew D. Pinto, Effie Gournis, Dana Al-Bargash, Rita Shahin

**Affiliations:** 1 Department of Family and Community Medicine, St. Michael's Hospital, Toronto, Canada; 2 Centre for Research on Inner City Health, Keenan Research Centre, Li Ka Shing Knowledge Institute, St. Michael's Hospital, Toronto, Canada; 3 Dalla Lana School of Public Health, University of Toronto, Toronto, Canada; 4 Toronto Public Health, Toronto, Canada; University of Lausanne, Switzerland

## Abstract

**Introduction:**

Labour disruptions that interrupt services can be a natural experiment to examine the effect of halting a program. A five-week municipal labour disruption in Toronto during the summer of 2009 provided an opportunity to investigate the impact of reduced sexual health services.

**Methods:**

We examined the incidence of reported chlamydia in Toronto during the five years (2004–2008) preceding the labour disruption and during the periods just before, during, and after the labour disruption. Comparisons of actual reports for 2009 were made to immediately adjacent periods around the labour disruption, to historical trends and to forecasted rates. Interrupted time series analysis was used to test for significant differences in the trend of reported chlamydia incidence.

**Results:**

There was no significant difference in the trend of reported chlamydia incidence around the time of the strike. However, there was a small but significant increase in the incidence of reported chlamydia, particularly among females under 25 years old immediately following the labour disruption. The reported incidence for this group was higher than would be expected based on annual increases and projected seasonal trends.

**Conclusions:**

There was a small increase in incidence of reported cases of chlamydia for certain groups that went beyond what is expected during the time immediately following the labour disruption. While causation cannot be implied from our ecological study, public health services may play a role in the control of sexually transmitted infections, even in the short-term. This underscores the need for future work to understand whether the changes observed can be attributed to the absence of these services.

## Introduction

Quantifying the impact of public health interventions is challenging. [1] Such interventions typically occur in complex environments, have multiple components and may require substantial time for changes to be observed. [2] An indirect way to evaluate an intervention is observing what happens in its absence. It would be unethical to halt the delivery of programs for research purposes, but natural experiments can be studied. [3] Such an event occurred in 2009 when a municipal labour disruption involving unionized staff at Toronto' public health unit occurred.

Evaluating the impact of health provider labour disruptions on health-related outcomes has precedence. Strikes by nurses in Canada, [4] England, [5] France, [6] and Sweden, [7] have been examined, as have those involving hospital housekeeping staff. [8] The impact of physician strikes and slowdowns on emergency room use and mortality has been explored in Croatia, [9] Israel, [10]–[14] and Spain. [15] Studies have also been conducted to examine the disruption of health services by natural disasters or armed conflict. [16] Interestingly, most studies found that mortality rates were unchanged or decreased when physician services were suspended. [17] While these ecological studies cannot prove causation, they identify interesting associations warranting closer examination of the impact of withdrawal or reduction of services on health outcomes. A review of the literature found no studies have yet been published on the impact of labour disruptions involving public health services.

In Ontario, public health is mandated to control STI through health promotion, screening, clinical services, distributing condoms, conducting surveillance and follow up of cases and contacts. [18] [19] Partner notification by health professionals has been shown to increase the detection of new infections [20], [21] as has partner counselling and referral services for HIV. [22], [23] A labour disruption affecting such services could theoretically lead to an increase in STIs through reduced access to diagnostic services, treatment and counselling, contact tracing, supplies (e.g. condoms) and this could lead to increases in primary and secondary infections.

This paper reports on the impact of the 2009 City of Toronto labour disruption on sexually transmitting infections (STI). We examine the association between the suspension of core STI public health services and the incidence of reported chlamydia. Chlamydia is by far the most common reportable STI in Toronto, and represented 58% of all diseases reported in 2009. [24] Infections result in significant costs to individuals and society. [25], [26] It is legally reportable to the Medical Officer of Health and has a short incubation period (7–14 days) [27] relative to the length of the labour disruption (36 days).

## Methods

### i. Setting

Toronto Public Health (TPH) is a large, urban health unit, with approximately 2,000 employees serving 2.6 million people. TPH operates a number of programs to address sexual health, including STI case management, sexual health clinics, health promotion campaigns, condom distribution, HIV/AIDS community engagement and a needle-exchange program. The STI case management program provides education, support and free medications to physicians for the treatment of STIs, counsels patients with an STI and performs partner notification.

Eleven sexual health clinics operate throughout the city. Five are solely managed by TPH, three in partnership with other organizations and three are funded but not operated by TPH. Health services are primarily directed to women under 25 years old and men under 30 years old as well as to those who otherwise experience barriers to accessing sexual health services. Clinic services include birth control and emergency contraception counselling and provision, pregnancy testing and counselling, free condoms and STI testing and treatment.

To estimate the proportion of STIs diagnosed in TPH supported clinics, all positive lab results for chlamydia, gonorrhea and infectious syphilis reported for people living in the TPH catchement area were examined. For 2010, the year closest to the labour disruption, 10.5% of reported chlamydia cases, 27.8% of reported gonorrhea cases and 35.5% of reported infectious syphilis cases were diagnosed in TPH supported clinics. [28] These numbers are likely underestimates of the population served in 2009, as some clients may have been lost to follow-up or chose to receive services elsewhere as a result of the 6-week closure. It should be noted that TPH is involved in the follow-up of all STI diagnosed in its jurisdiction, regardless of where the report originates.

### ii. Labour disruption and response

The normal delivery of services by TPH was disrupted for 36 days during the summer of 2009. Following six months of failed contract negotiations, Toronto municipal workers went on a legal strike from June 22 to July 27, 2009. [29], [30] All front-line employees of TPH were members of striking unions, which necessitated the reduction or cancelling of services. The sexual health program normally operates with 95 full-time equivalent (FTE) staff. This was reduced to five FTE non-unionized managers during the labour disruption. A contingency plan was activated to mitigate the impact of service disruption on population health. Priorities included the identification and prompt follow-up of cases deemed to be at high-risk of adverse health outcomes (new cases of chlamydia, gonorrhea or syphilis where the person is untreated, under 16 years old pregnant or HIV positive, and all new cases of HIV), responding to calls from the public and providing medications for STI treatment. Partner notification was suspended for almost all reported cases. All TPH-operated and partnership sexual health clinics were closed during the strike, and the public was redirected to other clinics. Harm reduction services, including the provision of condoms, safer injection supplies and safer crack kits, were suspended; however, a mobile van served a small number of clients. Harm reduction supplies, including condoms, were provided to partner agencies in the days leading up to the labour disruption. [31].

### iii. Data sources on chlamydia

Chlamydia is a notifiable disease under Ontario legislation. All confirmed cases of chlamydia (i.e. those with a positive lab report) with an episode date (the earliest date available on file, usually the date of symptom onset) between January 1, 2004 and December 31, 2009, living in Toronto at the time of diagnosis, were considered. [32] Case information was extracted from the integrated Public Health Information System (iPHIS) using Cognos Report Net (CRN) 1.0 on February 5, 2010. Annual incidence rates were calculated using Toronto population estimates for the public health unit provided by Statistics Canada, adjusted for census under-coverage and include non-permanent residents. Details on access to treatment and outcomes, including repeat testing and cure rates, were not available for analysis.

### iv. Classification of time periods and age groups

Based on the incubation period of chlamydia (7–14 days) and the dates of the labour disruption (June 22, 2009 to July 27, 2009), four time periods were defined (Table 1). The Period of Interest (POI) is the time when the population would be most likely to show the effects of the labour disruption, based on the typical chlamydia incubation period. Individuals infected with chlamydia during the labour disruption could begin to have symptoms as early as 7 days after the start of the disruption (i.e. June 29, 2009) and up to 14 days after the end of the labour disruption (August 10, 2009). This established our POI, which was 43 days long. For comparison, the same length of time was used to define the period before the disruption (Pre POI), after (POI+1), and two periods after (POI+2) the POI. We categorized cases as either 15–24 years old and 25+ years old in order to have roughly 50% of all cases in each age category.

**Table 1:** Time periods.

	Incubation period	Pre POI	POI	POI+1	POI+2
Chlamydia	7–14 days	May 17– June 28	June 29– August 10	August 11– September 22	September 23– November 4

*POI = Period of Interest.*

### v. Analysis

Statistical testing was carried out using SAS version 9.1 and Stata 12.1. For all analyses, comparisons with p<0.05 were considered statistically significant.

Simple linear regression was used to analyze annual trends. The MEANS procedure in SAS was run to calculate the corresponding confidence intervals. One sample *t*-tests were conducted to investigate the difference between the number of cases in 2009 and historical (2004 to 2008) averages. Trends between years were adjusted by using an adjusted rate for historical counts so they could be comparable to 2009 counts (Appendix). To compare consecutive intervals, the proportional change between time periods (i.e. change from Pre POI to POI, from POI to POI+1 and from POI+1 to POI+2) were calculated (Appendix). Rates of reported chlamydia in the absence of a labour disruption were estimated. These forecasted rates were calculated for the periods that included and preceded the labour disruption (POI, POI+1 and POI+2) for the two relevant age groups. The estimates assumed a worst-case scenario and approximated rates to the upper confidence limit of the adjusted historical counts. The upper confidence limit was chosen as a conservative estimate of forecasted rates of infection.

To further examine changes in the rates of reported chlamydia in 2009 before, during and after the labour disruption, an interrupted time series analysis was conducted. We examined four separate time series: males 15–24 years, males 25+ years, females 15–24 years and females 25+ years. The daily population adjusted incidence rates for each group was graphed against time between May 17, 2009 and November 4, 2009 and significant autocorrelation and non-stationary was suspected based on a visual inspection of the plot. This was confirmed when using the autocorrelation function (ac) in Stata. To achieve stationarity, we calculated moving averages at progressively larger windows, beginning at one. In each of the four time series, stationarity was achieved at a moving average window of seven, and this was confirmed through plotting correlograms and partial correlograms of the now smoothed time series data. Augmented Dickey-Fuller tests were conducted at lags one through five, and confirmed that there was no unit root in any time series. The correlograms suggested that autocorrelation would be corrected at lags higher than four, using Bartlett's formula. We developed separate auto-regressive, integrated, moving average (ARIMA) regression models for each group, using an auto-regressive component of four. We used the start of each period as an interruption point (i.e. June 29, August 11 and September 23), and included time, time after each intervention, and the historical average incidence (based on the daily incidence from 2004–2008) into the regression formula. To test the appropriateness of this model we examined whether the lag component was significant and examined the residuals for normality and whether they were distributed around zero.

## Results

**Figure 1:**
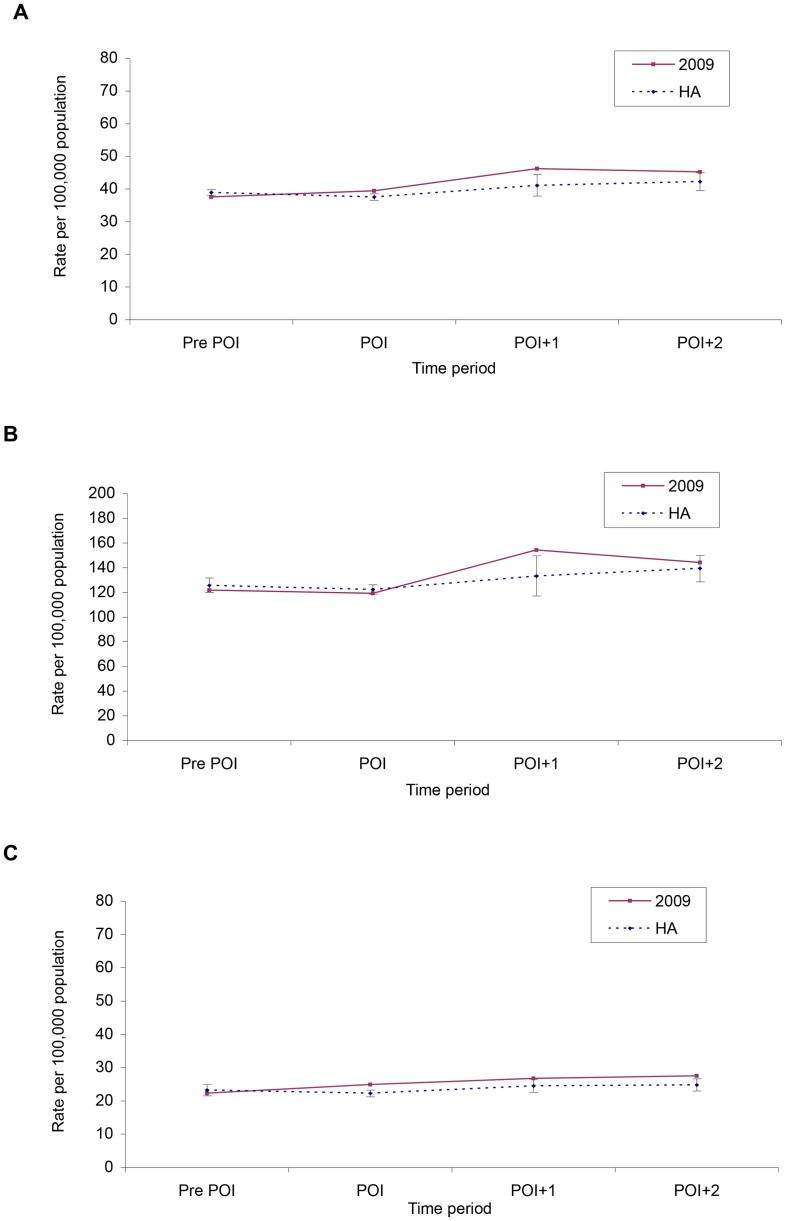
Incidence (rate per 100,000) of reported chlamydia by time period for 2009 and adjusted 2004–2008 historical average (HA) for A. all cases (15+ years), B. persons 15–24 years old and C. persons 25 years and older. Error bars indicate 95% confidence intervals.

A total of 41,965 cases of chlamydia were reported to Toronto Public Health from 2004 to 2009. Approximately 50% of chlamydia cases in 2009 were in persons aged 15–24 years. There was a significant, linear increase in annual rates of reported chlamydia, ranging from 3–8% increase per year from 2004 to 2009 (p<0.01). The 2009 rates were significantly higher than the historical average (p<0.01) (Table 2). The reported chlamydia incidence rate for the Pre POI period was significantly lower than the comparable adjusted historical rate for the same period (p = 0.02). This trend changed for the POI and POI+1 periods when the rates were significantly higher than the comparable adjusted historical periods (p = 0.01 and p = 0.02, respectively) (Figure 1A).

**Table 2:** Reported cases of chlamydia in 2009 compared to the historical average (2004–2008).

	Historical average	2009
Number of reported cases of chlamydia	6803	7951*
Incidence rate of reported cases of chlamydia (per 100,000)		
Male	210.6	234.0*
Female	306.2	362.1*
All	259.8	299.8*

* *Significantly different* (α = 0.05) *from historical average*.

Time trend analysis by age group for chlamydia revealed that both 15–24 and 25+ age groups had increased reported rates in 2009. The 15–24 age group had a significant increase from the adjusted historical average during the POI+1 period (p = 0.03) (Figure 1B), while the 25+ group had a significant increase over historical reported rates during the POI (p = 0.009) (Figure 1C).

For all reported chlamydia cases, the proportional change from the Pre POI to POI in 2009 was significantly higher than comparable historical changes (p = 0.01) (Table 3). For specific age groups, significant differences between these two periods were noted only for the 25+ years old group (p = 0.01). When examining these changes by gender, this increase in incidence occurred for males but not females. The 15–24 years old age group only showed a significant increase in 2009 from the historical changes from the POI to POI+1 period (p = 0.01), which was not detected when examining the change for all reported chlamydia cases during those periods. Further, this increase was seen in young females, but not older females (25+ years old) or males.

**Table 3:** Percent change between consecutive time periods for reported chlamydia, by age and gender group for 2009 and 2004–2008 historical average (HA).

		Pre POI to POI			POI to POI+1			POI+1 to POI+2		
	Age Group	2009	HA	p-value	2009	HA	p-value	2009	HA	p-value
*ALL*	**15–24**	−2.1%	−2.7%	0.64	29.3%	8.9%	0.01*	−6.4%	5.7%	0.15
	**25+**	11.9%	−3.8%	0.01*	7.3%	10.3%	0.36	2.9%	1.5%	0.80
	**Total**	4.9%	−3.4%	0.01*	17.3%	9.6%	0.06	−2.2%	3.3%	0.32
*MALE*	**15–24**	7.1%	−3.4%	0.04*	20.0%	7.2%	0.06	3.5%	5.2%	0.86
	**25+**	16.1%	−4.0%	0.01*	7.1%	8.5%	0.77	0.4%	2.7%	0.72
	**Total**	12.7%	−3.9%	0.01*	11.3%	7.9%	0.22	1.6%	3.1%	0.75
*FEMALE*	**15–24**	−5.9%	−2.0%	0.24	32.9%	9.9%	0.01*	−9.6%	6.3%	0.07
	**25+**	8.0%	−3.5%	0.05	7.0%	12.4%	0.07	5.1%	1.1%	0.63
	**Total**	0.2%	−2.8%	0.26	20.8%	10.9%	0.05	−4.1%	3.8%	0.29

* *Significantly different* (α = 0.05) *from historical average.*

*POI = Period of Interest. HA = Historical Average.*

**Figure 2:**
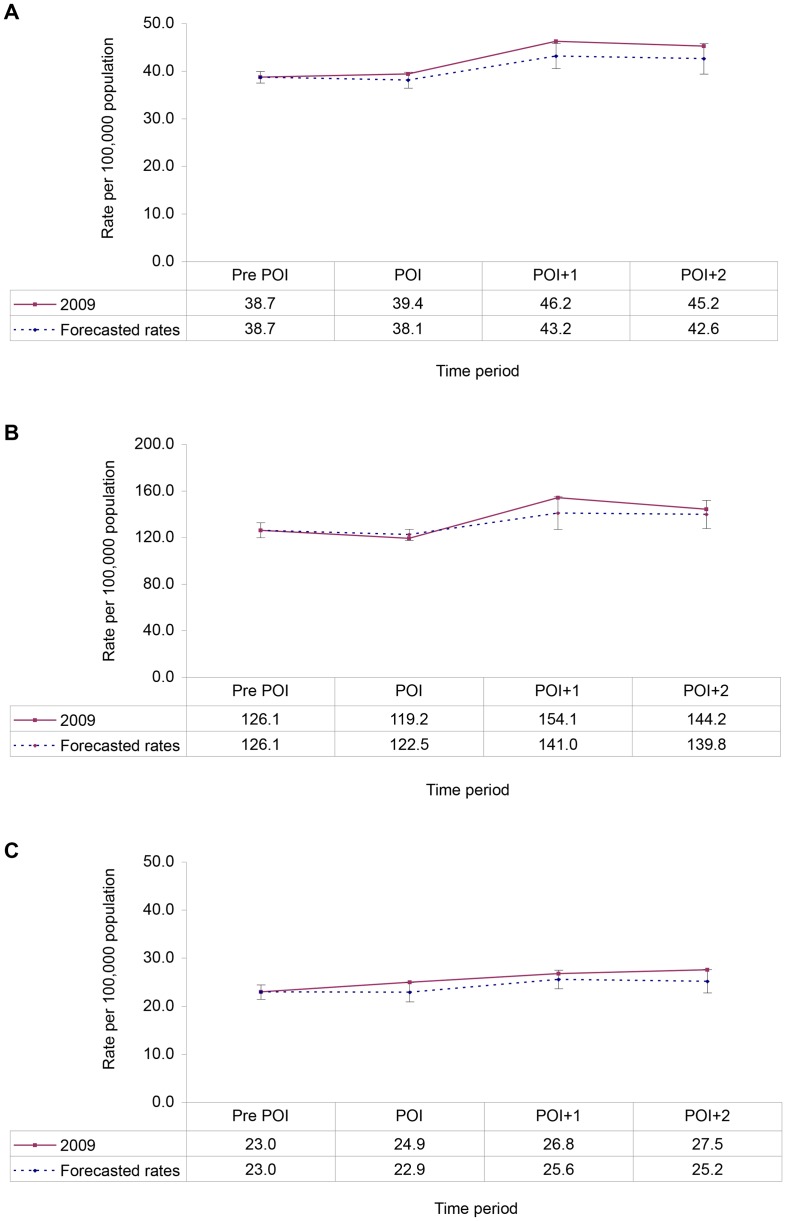
Forecasted rates versus actual incidence rate of reported chlamydia by time period for 2009, for A. all cases (15+ years), B. persons 15–24 years old and C. persons 25 years and older. Error bars indicate 95% confidence intervals.

A comparison to forecasted data with actual incidence rates indicates there was a higher rate of reported chlamydia during the POI, and POI+1 periods than expected (Figure 2A), which was particularly pronounced during the POI+1 for those aged 15–24 years old (Figure 2B), but not in those aged 25 years and older (Figure 2C).

The interrupted time series analysis tested whether there were significant differences in the incidence and trend in reported chlamydia rates for each age group and gender between specified periods. The residuals for each model were normally distributed and centred around zero. The ARIMA model using a lag 4 was not significant for males 15–24 (p = 0.123), but was for males 25+ (p = 0.015), females 15–24 years (p = 0.018) and females 25+ (p = 0.015). As shown in Table 4, time was a significant predictor of the incidence of chlamydia in all groups. For males aged 15–24 and aged 25+, there was a small significant decrease in incidence in the POI. However, this is largest for men aged 15–24, but is questionable as the ARIMA model at lag 4 was not significant. The most noteworthy finding is that there was an increase in incidence (coefficient 1.811, p<0.001) for females aged 15–24 in the POI+1 period, confirming the findings from the previous analysis.

**Table 4:** Results from autoregressive integrated moving average models (ARIMA) developed for different age and gender groups.

	Males						Females					
	15–24			25+			15–24			25+		
	Coefficient	95% CI	p-value	Coefficient	95% CI	p-value	Coefficient	95% CI	p-value	Coefficient	95% CI	p-value
**Constant**	1.260	(0.985, 1.535)	<0.001*	0.423	(0.343, 0.502)	<0.001*	2.927	(2.398, 3.457)	<0.001*	0.369	(0.306, 0.431)	<0.001*
**Time**	0.012	(0.001, 0.022)	0.027*	0.002	(0.000, 0.005)	0.027*	0.036	(0.016, 0.057)	<0.001*	0.007	(0.005, 0.009)	<0.001*
**POI (June 29)**	−0.576	(−0.998, −0.154)	0.007*	−0.097	(−0.177, −0.017)	0.018*	−0.750	(−1.598, 0.098)	0.083	−0.136	(−0.273, 0.002)	0.053
**Time after POI**	0.004	(−0.011, 0.020)	0.587	0.001	(−0.003, 0.005)	0.626	−0.042	(−0.075, −0.009)	0.014*	−0.005	(−0.009, −0.001)	0.015*
**POI+1 (August 11)**	0.015	(−0.507, 0.537)	0.955	−0.031	(−0.172, 0.110)	0.664	1.811	(0.937, 2.685)	<0.001*	0.000	(−0.086, 0.085)	0.992
**Time after POI+1**	−0.015	(−0.036, 0.005)	0.14	−0.003	(−0.007, 0.002)	0.259	−0.011	(−0.045, 0.023)	0.540	−0.002	(−0.007, 0.002)	0.308
**POI+2 (September 23)**	0.115	(−0.296, 0.526)	0.584	0.030	(−0.084, 0.145)	0.606	−0.521	(−1.539, 0.496)	0.315	0.096	(−0.017, 0.208)	0.096
**Time after POI+2**	0.000	(−0.019, 0.019)	0.982	−0.002	(−0.007, 0.003)	0.519	0.031	(−0.058, 0.187)	0.154	−0.001	(−0.006, 0.004)	0.739
**Historical average**	−0.022	(−0.135, 0.090)	0.696	0.053	(−0.077, 0.182)	0.424	0.065	(2.398, 3.457)	0.300	0.019	(−0.105, 0.143)	0.760

* *Significant* (α = 0.05).

## Discussion

This study attempts to estimate the impact of a labour disruption involving public health services, focusing on new reported chlamydia infections. While any association between changes in disease incidence before and after a labour disruption is purely ecological, this study did show there were observable differences. We found a higher incidence of reported cases of chlamydia than would be expected in the POI for the 25+ age group and in the POI+1 for the 15–24 year age group. The greatest changes between periods of interest were seen in the female 15–24 age groups from the POI to POI+1 and this change was higher than the historical trend.

It is difficult to explain the differences observed between the genders and age groups in the timing of the increase in reported chlamydia infections. Men and women may be tested for different reasons, with women being more likely to be screened when asymptomatic than men. Information on the reason for testing was not available. For older persons, the increase in incidence was seen earlier (during the POI) than than for younger persons (during the POI+1). Older persons may be more likely to recognize the symptoms of chlamydia as an STI and seek care immediately. However, this likely explains only part of this difference, as chlamydia is often asymptomatic. Younger persons may be less likely to seek STI testing after engaging in high-risk sexual behavior (e.g. unprotected sex), and hence may present at a time later in in their transmission than older persons. Finally, the age differences seen may reflect that TPH programs, particularly sexual health promotion and sexual health clinic services, are targeted to younger groups and the absence of these services would most likley be experienced in this age group. Older persons may also have greater access to other health care services such as family physicians, that were not affected by the labour disruption. Such access could mean testing could occur earlier and the date of infection be attributed to an earlier date. Age may also play a role in the number of partners, the notification of partners by infected individuals and the use of condoms, all of which may affect the rate of STIs in those 25 years and older. The differences in timing for the two age groups may relate to the older group being most affected by a lack of safe sex supplies provided by public health and the younger age group being most affected by lack of access to treatment. There is also the possibility that the increases in the older age group are unrelated to the labour disruption all together and reflect some other unmeasured factor.

Several important limitations to this study should be noted. First, it is impossible to definitively attribute any change to the burden of disease to the specific provision or absence of public health services, given that this is an observational study. Many other factors could lead to the observed increase in the incidence of chalmydia. [33] For example, over a long period of time there may be changes in the characteristics of the pathogen, although this is very unlikely over the time span of our study. [34] Increased availability and uptake of non-invasive nucleic acid amplication tests have been hypothesized as key to witnessed increase in incidence in chlamydia since the 1990s, but again, this is unlikely to have changed significantly over the study period. A large scale public event or mass gathering during the summer of 2009 could lead to an increase in STI transmission, although the evidence for this happening in other contexts is sparse. [35] Changes in the accessibility of other health services and the social determinants of population health can certainly play a role in the incidence of STI. [36].

As noted above, TPH supported clinics represents a small part of the health care system where patients are tested for STI, and we were unable to evaluate whether patients sought care elsewhere or deferred care. We used the reported episode date, which is an extrapolation to a potential date of infection as it is most often based on symptom onset or at worst, specimen collection date. Dividing up time periods based on incubation periods may not account for the large variability in estimates of the incubation period. We assumed a close temporal association between acquiring an STI, getting tested and getting treated. However, many STIs, including chlamydia, can be asymptomatic and may be detected as latent infections. [27] The increase in incidence may reflect deferred care-seeking by clients who acquired infection at an earlier time. However, this would have been adjusted for if symptom onset date was consistently obtained for these cases and reflected in the episode date used. Finally, we do not know if those using TPH services are representative of the general population.

This was a unique study examining the impact of a sudden reduction in the availability of sexual health services on the incidence of reported cases of chlamydia. We found a small but significant an association between the disruption of public health services and health outcomes. Future studies could examine other STIs and how such labour disruptions impact the number of people seen by primary care providers or non-TPH STI clinics. This could be accomplished by surveying individuals infected with an STI about their health seeking behaviour during such a disruption, or by using administrative data such as health insurance billing information. It would be of value to conduct this type of study prospectively and incorporate it into the evaluation of public health services. Public health can make use of such natural experiments to evaluate its impact on health outcomes at a population level.
